# Synthesis of Transparent CuI Thin Films by a Facile Low‐Cost High Pressure (HP)‐PECVD Method at Room Temperature for the Application in Solar Cells

**DOI:** 10.1002/open.202300067

**Published:** 2023-09-12

**Authors:** Arindam Sanyal Dipto, Liton Mondal, Jaker Hossain, M. Mamunur Rashid, M. Khalid Hossain, Nepal C. Roy, Mamunur Rashid Talukder

**Affiliations:** ^1^ Plasma Science and Technology Lab Department of Electrical and Electronic Engineering University of Rajshahi Rajshahi 6205 Bangladesh; ^2^ Plasma-processed Functional Materials Laboratory Department of Electrical and Electronic Engineering University of Rajshahi Rajshahi 6205 Bangladesh; ^3^ Atomic Energy Research Establishment Bangladesh Atomic Energy Commission Dhaka 1349 Bangladesh; ^4^ Chimie des Interactions Plasma-Surface (ChIPS) CIRMAP, Université de Mons 23 Place du Parc 7000 Mons Belgium

**Keywords:** band gap, CH_3_CN, CuI thin film, HP-PECVD, transparent

## Abstract

Copper iodide (CuI) thin films were prepared on a glass substrate by a facile high pressure (HP)‐PECVD method at room temperature. For this, CuI powder was dissolved in CH_3_CN. The CuI vapor with plasma was investigated by Optical Emission Spectroscopic (OES) data for identifying the species in the plasma. The XRD study reveals the polycrystalline nature of the films. The SEM analyses indicate the homogeneity of the films. The EDS mapping confirms that the thin films mostly consisted of carbon followed by nitrogen, copper and iodine, respectively. The band gaps of CuI thin films were in the range of ~2.71–3.14 eV. The high transmittance and band gap engineering in HP‐PECVD‐synthesized CuI thin films indicates their potential use as window and hole transport layers in low cost solar cells.

## Graphical abstract

CuI thin films have been deposited on a glass substrate by a low cost HP‐PECVD method. The CuI vapor has been produced by dissolving CuI powder in CH_3_CN solvent. CuI plasma has been analyzed using Optical Emission Spectroscopic (OES). XRD study reveals the polycrystalline nature of the CuI films, while SEM analyses indicate the homogeneity of the films. The band gaps of CuI thin films are in the range of 2.71–3.14 eV.

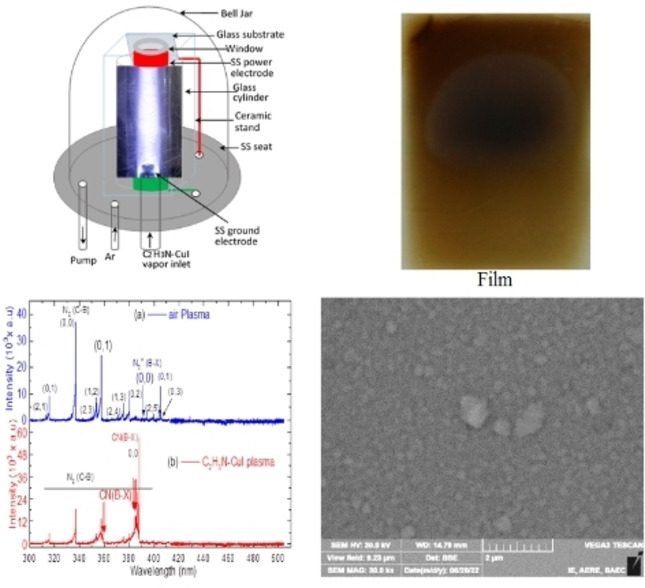

## Introduction

Uniform and ultra‐thin coatings play important roles for manufacturing electronic devices and consequently the demands of such thin films are of immense importance for electronic device manufacturing industries. Therefore, development of affordable and cost‐effective thin film deposition system is an important research area. The zinc blende copper iodide (CuI), one of the most important electronic materials, is a wide band gap highly transparent native p‐type binary compound semiconductor.[^1^, ^2^] The direct band gap of CuI thin films is 3.1 eV and shows a high transparency >80 %, hole mobility >40 cm^2^ V^−1^ s^−1^, and conductivity >150 S ⋅ cm^−1^.[^1^, ^2^, ^3^, ^4^, ^5^] The high p‐type conductivity of CuI thin film arises due to small hole effective mass (0.3 m_0_) and its conductivity can further be improved by iodine doping that plays a role of electron acceptor creating holes in the valance band.[^2^, ^3^, ^6^] CuI thin films have a wide range of applications such as CuI electrode in organic electronics, thermoelectric, conductor of holes in CuI/n−Si heterojunction, organic, polymer and perovskite solar cells.[^7^, ^8^, ^9^, ^10^, ^11^, ^12^, ^13^, ^14^]

Literature reports indicate that different techniques have been deployed for the deposition of CuI thin films, for example, reactive sputtering, thermal evaporation, spin coating, drop‐casting, vapor‐phase iodination, spray pyrolysis, epitaxy, chemical bath deposition (CBD) and successive ionic layer adsorption and reaction (SILAR) methods.[^1^, ^2^, ^15^, ^16^, ^17^, ^18^, ^19^, ^20^, ^21^, ^22^, ^23^, ^24^] In the case of reactive sputtering, the room‐temperature‐synthesized I‐doped CuI films show high conductivity with surface homogeneity.^[1]^ The solid phase iodination of Cu or Cu_3_N results in thin films that have higher homogeneity and transparency with high mobility than the films prepared by conventional methods.^[18]^ On the other hand, the CuI thin films produced by epitaxy show superior rectification properties in heterostructures.^[20]^ CuI thin films prepared by the CBD method show more surface smoothness than the SILAR method.^[22]^ However, among the methods, the spin coating is a very simple, low temperature and low‐cost technique for the fabrication of thin films. Recent work on liquid phase iodination in spin‐coated CuI thin films shows a potential increase in conductance of the films to be used as a hole transport layer.^[2]^ However, film discontinuity and roughness are very high, although the liquid phase iodine doping slightly decreases the roughness and thereby enhances the carrier transport in the CuI thin films. Therefore, an alternative low‐cost solution process deposition technique is indispensable for the deposition of CuI thin films. In this endeavor, we demonstrate the fabrication of CuI thin films by a low cost high‐pressure plasma‐enhanced chemical vapor deposition (HP‐PECVD) method for the low cost application in solar cells.

Traditional low‐pressure (LP) systems such as CVD,^[25]^ PECVD,^[26]^ DC magnetron sputtering,^[27]^ HiPIMS,^[28]^ ALD,^[29]^ and others, are operated in the mTorr pressure range^[30]^ and, therefore, they need costly RF sources and high vacuum systems. In spite of facing a set of challenges, plasma assisted thin film deposition systems receive a lot of attention for the development of thin films that are highly uniform, pinhole‐free along with reduced roughness. However, in order to have thin film properties of newly developed HP‐PECVD deposition system comparable to that of LP‐PECVD methods, a lot of tunings are needed, for instance, electrode geometry, sample injection technique, reactor pressure, source power and frequency because the plasma properties are highly influenced by pressure and operating frequency.^[31]^ It is observed that a number of methods for the film deposition at high pressure employing different types of plasma sources due to their low cost because there is no need of expensive vacuum system along with relevant accessories. For instances, the Reniers group^[32]^ have showed a deposition method with an atmospheric pressure (760 Torr, AP) dielectric barrier discharge (DBD), that is, AP‐DBD plasma system, where they manipulated filamentary mode DBD plasma and which can be exploited for thin film or coating preparation, but it could be of interest only for special object‐oriented approaches. The glow discharges possess uniform plasmas because of the absence of streamers that can be easily generated by reducing pressure for the deposition of uniform film. Recently, Ussenov et al.^[33]^ have studied a hybrid plasma system (plasma jet along with spark discharge) for the deposition of CuO_x_ thin films. Further, Tsai et al.^[34]^ have explored the properties of conductive Cu films prepared with AP‐PECVD He/H_2_ plasma jet in ambient air from Cu(II) acetylacetonate vapor. The major drawbacks of these Cu‐based films prepared by this AP plasma system are the discontinuity of the films as well as strong film thickness inhomogeneity.

This study explores the preparation of CuI films using a simple and low‐cost HP‐PECVD system considering the importance of CuI thin films in versatile applications. The study includes diagnostics of plasma system, such as plasma species produced, power dissipated and gas temperature along with probable chemical kinetic pathways in the discharge. The synthesized thin films were characterized by XRD, SEM, FTIR and UV‐vis spectroscopy. The high transmittance and band gap engineering with deposition time, that is, thickness of the synthesized CuI thin films, reveal the potential applications of the semiconductor as window and hole transport layers in low cost silicon and perovskite photovoltaics.

## Experimental Section

### Materials and preparation of CuI thin films

The CuI powder, and acetonitrile (CH_3_CN) chemicals were acquired from Sigma‐Aldrich. The CuI powder was added in CH_3_CN solvent with a concentration of 1 mg/mL and mixed by a magnetic stirrer. Then the resultant solution was filtered using a nylon filter having a pore diameter of 0.45 μm. The glass substrates were first cleaned by piranha solution to dispel the gross contaminants. Then they were cleaned by acetone and distilled water and dried by air blower prior to film deposition. The glass substrates were placed within the substrate holder of the HP‐PECVD deposition system and then CuI thin films were deposited at room temperature.

**Figure 1:**
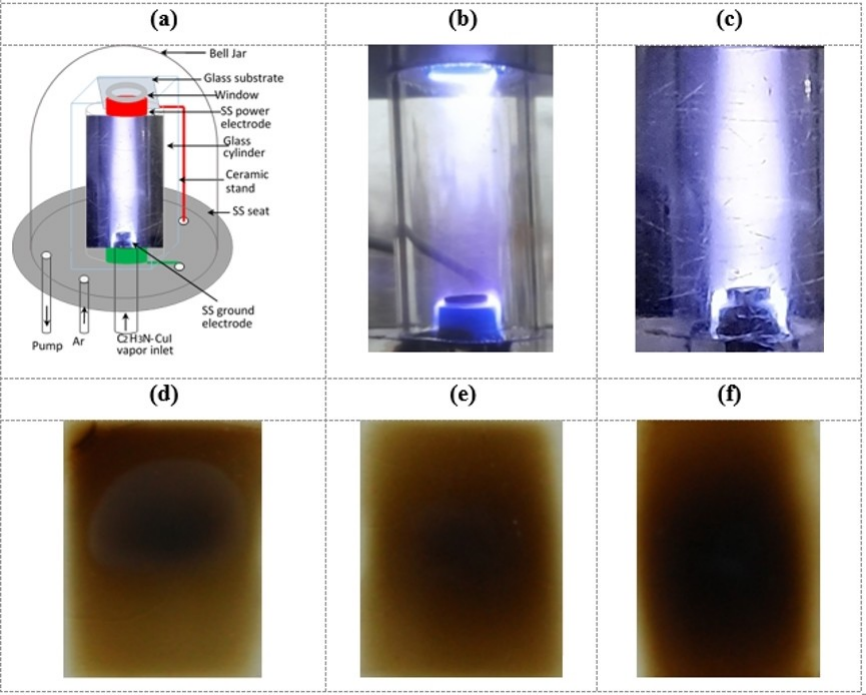
(a) Schematic of the high pressure plasma enhanced chemical vapor deposition (HP‐PECVD) system used for CuI thin film preparation, photographs of glow discharges in (b) air, and (c) $CH_{3}CN-CuI$ and deposited films for deposition time of (d) 5, (e) 7, and (f) 10 min.

Figure 1a shows the schematic of the experimental setup used for the preparation of CuI thin films and discharge images along with prepared CuI films. The setup contains vacuum chamber, rotary pump, high voltage power supply, high voltage and current probe, digital oscilloscope, two spectrophotometers, ceramic stand and substrate holder, and cylindrical glass tube. The CuI solution was kept in a conical flask whose outlet was connected to the deposition chamber by a flexible silicon tube. There were five orifices at the steel seat and used as inlet and outlet ports. Two side‐orifices were used for connecting rotary pump for lowering inside pressure of the bell jar and gas inlet. High voltage wires were inserted through the remaining two side‐orifices. A Pyrex glass tube was inserted through the center orifice and was used as C_2_H_3_N−CuI vapor inlet channel. One of the two ring‐shaped (height 10 mm and ring inner diameter 9 mm) stainless steel electrodes were used as high voltage electrodes, as shown in Figure 1. A power electrode was placed at the top of the ceramic stand while the grounded electrode was placed at the bottom. Pyrex glass substrate (40 mm×25 mm×1 mm) was placed at the axial center of two electrodes as well as on the upper surface of the power electrode. Distance between the two electrodes was 58 mm. A Pyrex cylindrical glass tube (height 58 mm, inner diameter 18 mm) was used both as active plasma discharge channel as well as CH_3_CN−CuI vapor flow channel, where physicochemical reaction took place. There was a circular window (diameter 12 mm) at the top ceramic base plate in order to penetrate CuI solution for the deposition of film.

The glass substrate was placed at the substrate holder and the setup was enclosed with bell jar. A rotary pump was used to reduce inside pressure of the bell jar and the pump was allowed to operate during whole film preparation period. A 15 kV, 50 Hz power supply was connected across the electrodes and plasma was produced between the electrodes enclosed with cylindrical Pyrex glass tube used for the confinement of plasma within the glass tube as shown in Figure 1b. Inside pressure of bell jar was maintained at 60 Torr. C_2_H_3_N−CuI solution was started to evaporate due to opening the stop cock of the container because of lowering inside pressure (~60 Torr) of the container and the solution was evaporated at a rate of 0.75 mL/min. Subsequently, the plasma discharge color was changed due to the presence of C_2_H_3_N−CuI vapor in the discharge zone as shown in Figure 1c. Room temperature deposition means no additional heat was supplied to deposition system for the film deposition.

Figures 1d–f show the optical images of CuI thin films deposited for various time by HP‐PECVD method. It is seen from the Figures that the color of the films deepened with deposition time which happened owing to the increase of the films thickness with time.

**Figure 2:**
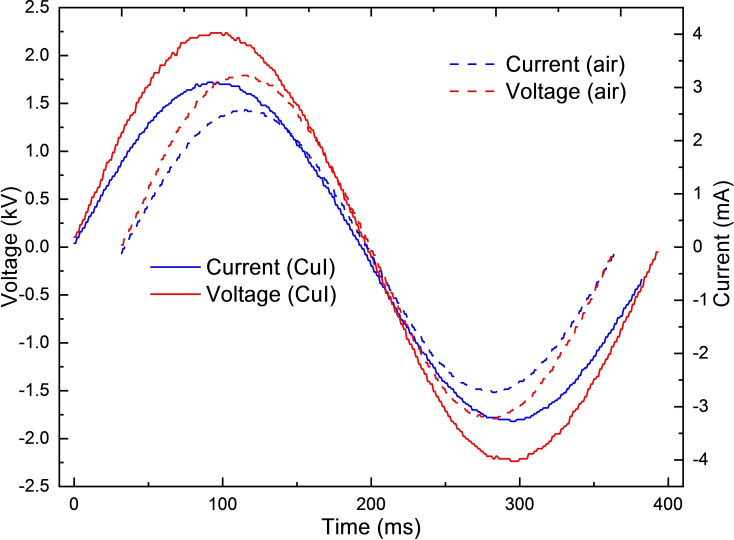
Voltage‐current characteristics of the glow air and $CH_{3}CN-CuI$ discharges.

Discharge voltage and current were measured with a high voltage and current probe in combination of an oscilloscope in order to determine plasma discharge power in HP‐PECVD process as shown in Figure 2. The HP‐PECVD voltage and current were measured with high voltage probe (HVP‐08) and current probe (CP‐07 C) along with a digital storage oscilloscope (RIGOL DS1104). The electrical power () dissipated in the discharge was determine by integrating the voltage () and current () over a period () using the following Equation 1:

(1) $$w=\left(\frac{1}{T}\right)\int_{0}^{T}v\left(t\right)i\left(t\right)dt.$$

Besides, a high resolution spectrophotometer (Avantes, Avaspec 2018, detector range: 200–500 nm, slit width 10 μm, 2400 lines/mm, optical resolution 0.07 nm) was used for the determination of rotational and vibrational temperatures as well as the identifications of species produced in the discharge. A thermocouple was used to determine the substrate temperature under different experimental conditions, in order to correlate the film properties with the substrate temperature.

### Thin film characterization

The thicknesses of the CuI thin films were estimated by a thickness profiler of model Bruker Dektak XTL. The average deposition rate of HP‐PECVD deposited CuI thin films was measured to be ~24.5 nm/min. The thicknesses of the CuI thin film were 125, 165 and 250 nm for deposition time of 5, 7 and 10 min, relatively. The generic characteristics of the films were explored by thickness, optical properties, X‐ray diffraction characterization, and surface morphology by scanning electron microscope (SEM) and Fourier transform infrared (FTIR) spectroscope for the identification of chemical bonds. The transmittance and absorbance data were obtained by UV‐VIS spectrophotometer (UV‐1900i, Shimadzu Corporation, Japan). The XRD data were taken by XRD Diffractometer (X'Pert‐Pro, Philips, Japan) with the monochromatic CuK_α_ radiation (λ=1.54056 Å). The SEM and EDS studies were carried out by the SEM systems of Carl Zeiss (Model: EVO‐18 Research).

## Results and Discussion

### Plasma Diagnostics

Plasma properties play important roles in the production of plasma species through different collision mechanisms, such as electron impact excitation, ionization, dissociations and attachment of atoms and molecules in the HP‐PECVD system. In order to understand the physicochemical processes involved in the HP‐PECVD plasma system and subsequently film deposition mechanism, power dissipated in the discharge, gas temperature, substrate temperature, species produced in the discharge were determined and identified. Therefore, plasma diagnostics were performed in two steps i. e. electrical and optical diagnostics.

**Figure 3:**
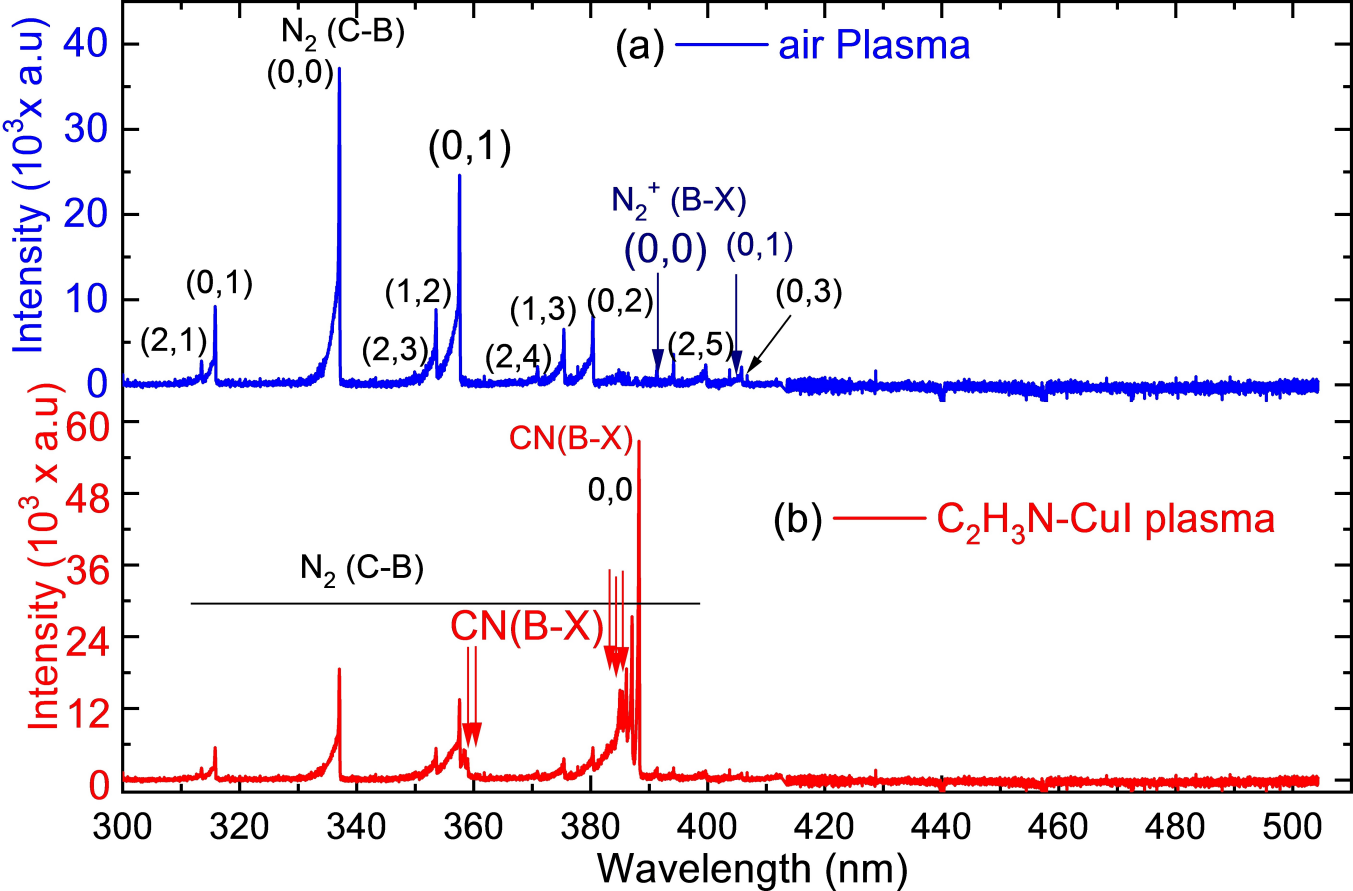
Optical emission spectra of (a) air and (b) $CH_{3}CN-CuI$ glow discharge plasmas (flow rate: $0.75\;\;mL•{min}^{-1}$ , electrode gap: 58mm , discharge voltage: ∼5kV , and frequency: 50Hz ).

Measured discharge voltage and current were used for the determination of dissipated power using Equation (1). The power consumption of the plasma was 7.83 W for air and 13.60 W for C_2_H_3_N−CuI vapor in HP‐PECVD plasmas. Optical emission spectrum (OES), shown in Figure 3, shows the species produced in air and CuI discharges.

**Figure 4:**
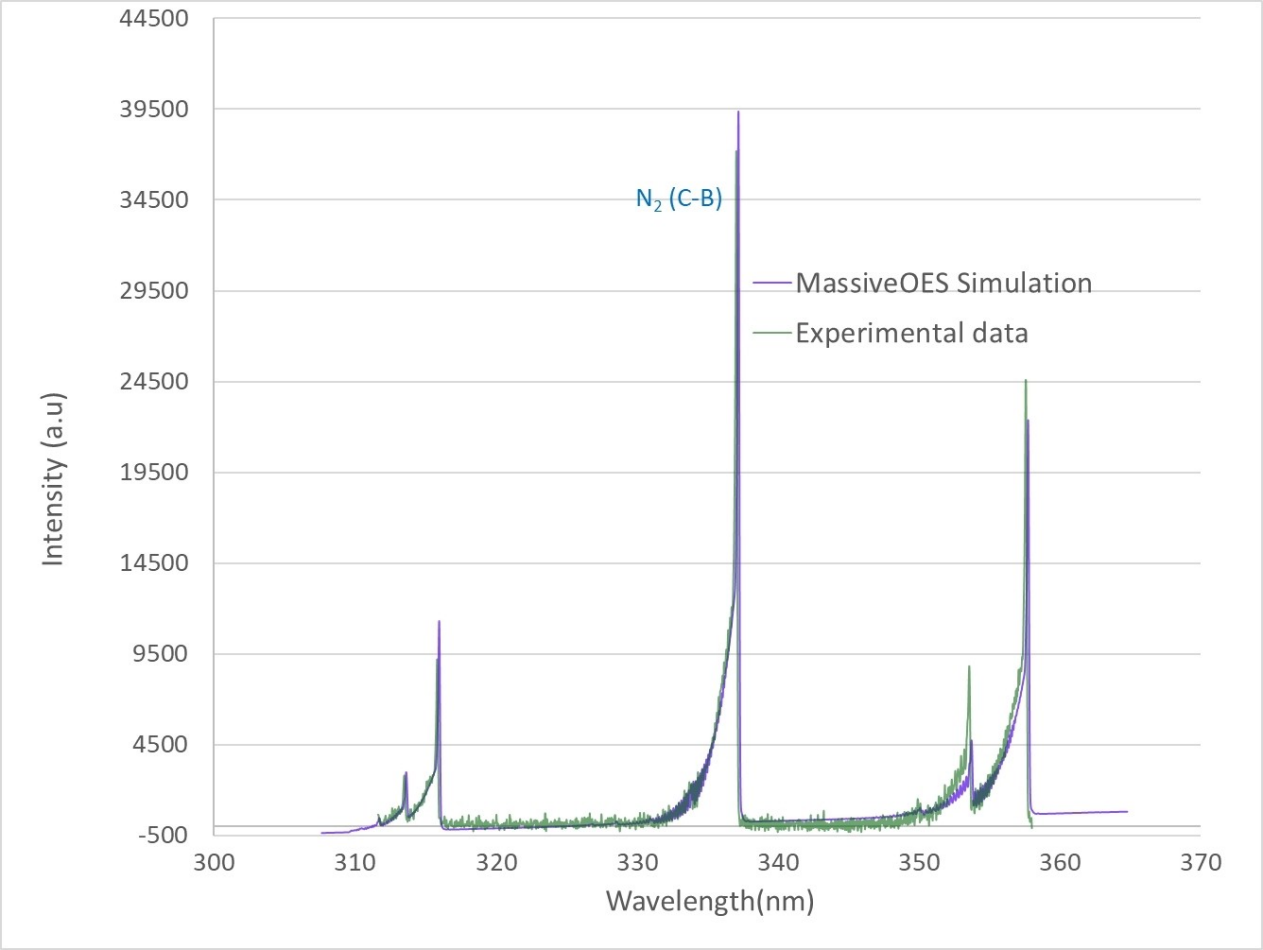
The experimental and fitted data of $N_{2}\left(C-B\right)$ band to determine rotational temperature ($T_{r}$ ) and vibrational temperature ($T_{v}$ ) of $CH_{3}CN-CuI$ plasma.

The OES data were also fitted with massive OES software^[35]^ to determine rotational ($T_{r}$ ) and vibrational ($T_{v}$ ) temperatures of the heavy species as shown in Figure 4. Fitted data reveal that $T_{r}=1185\pm 34\;K$ and 1479±48K whereas, $T_{v}=2124\pm 76\;K$ and 2315±73K in air and CuI plasmas, respectively.

The appearance of the chemical bonds as well as the percentage of the elements on the thin film can be explained by the chemical kinetics in the plasma discharge. As the discharge medium contains ambient air since the reactor pressure (60 Torr) can be considered as high pressure, intense band of N_2_ second positive system (SPS) appeared in OES spectra (Figure 3).

The direct electron impact dissociation of CH_3_CN mainly lead to the formation of CN radicals as it appears as the highest peak in the OES spectrum. The followings are the major species that can be deduced from the product appeared as shown in Equations (2) to 6:

(2) $$CH_{3}CN+e\to CH_{3}+CN+e$$

(3) $$CH_{3}CN+N_{2}\left(A\right)\to CH_{3}+CN+N_{2}$$

(4) $$CN+HCN\to C_{2}N_{2}+H$$

(5) $$CN+e\to CN^{{}^{*}}+e\to C+N+e$$

(6) $$CN+CN\to C_{2}+N_{2}$$

It is worth noting, there is a limitation that OES can only detect excited species produced in plasmas, however, as CuI dissolved into CH_3_CN, the formation of Cu^2+^ is likely to be expected through natural chemical dissociation process. Nevertheless, the transition of particular element depends on the rate constant of the reaction concerned. On the other hand, the presence of liquid CH_3_CN may deexcite Cu through quenching mechanism, which could be one of the possibilities of the absence of Cu transition in OES spectra, but it does not mean that Cu is not activated or excited by plasma. The formation of CuI thin film mainly produced through reaction shown below in Equations (7) and 8:

(7) $$CH_{3}+I\to CH_{3}I$$

(8) $$CH_{3}I+Cu\to CH_{3}+CuI.$$

However, the elemental analysis of CuI thin films prepared by HP‐PECVD also reveals that the film contains C, N, Cu and I, respectively. The details of the composition are discussed later.

Inside rotational temperature (considered as gas temperature) of the HP‐PECV reactor is $T_{r}\approx 1479\;K$ . The structure of the CuI thin films depends on reactor temperature [36], such as it takes zinc blend structure (γ-CuI ) when the temperature stays below 663K , wurtzite structure (β-CuI ) in the temperature range $\left(663-713\right)\;K$ and cubic structure (α-CuI ) for the temperature above 713K . Therefore, one may consider that the CuI thin films can be of cubic structure because the gas temperature within the plasma discharge zone was ∼1479K . Besides, one can also consider that such high temperature may affect species kinetics within the reactor and the discussion concerning this phenomenon is beyond the scope of this work.

### XRD studies of HP‐ PECVD CuI thin films

**Figure 5:**
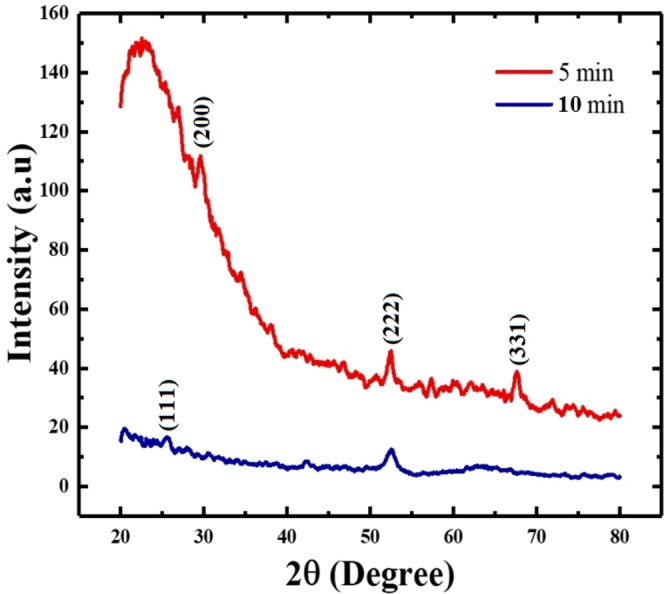
XRD spectra of HP‐PECVD deposited CuI thin films.

Figure 5 depicts the XRD spectra of CuI thin films deposited by HP‐PECVD method. It is observed from the figure that the film deposited for 5 min shows three weak peaks at 29.6, 52.5 and 67.6° those correspond to (200), (222) and (331) planes whereas the film deposited for 10 min shows two peaks at 25.6 and 52.5° those correspond to the (111) and (222) planes according to the ASTM ref. PDF 01‐076‐0207. These results indicate zinc blende face centered cubic γ‐CuI phase of the films.[^2^, ^37^, ^38^] Consequently, it can be inferred that CuI thin films deposited by HP‐PECVD technique using CH_3_CN solvent are polycrystalline in nature.

The size of crystallites were calculated from the Debye–Scherrer formula shown in Equation 9,^[39]^

(9) $$D=\frac{0.94\lambda }{\beta cos\theta }$$

where, λ denotes the X‐ray wavelength, β denotes the full width at half maximum (FWHM) in radians of different planes.

The crystallites size of the film deposited for 5 min with planes (200), (222) and (331) were 27.5, 30 and 31 nm whereas that for the 10 min film with (111) and (222) planes were 16.5 and 26 nm, respectively.

### Surface morphology of HP‐ PECVD CuI thin films

**Figure 6:**
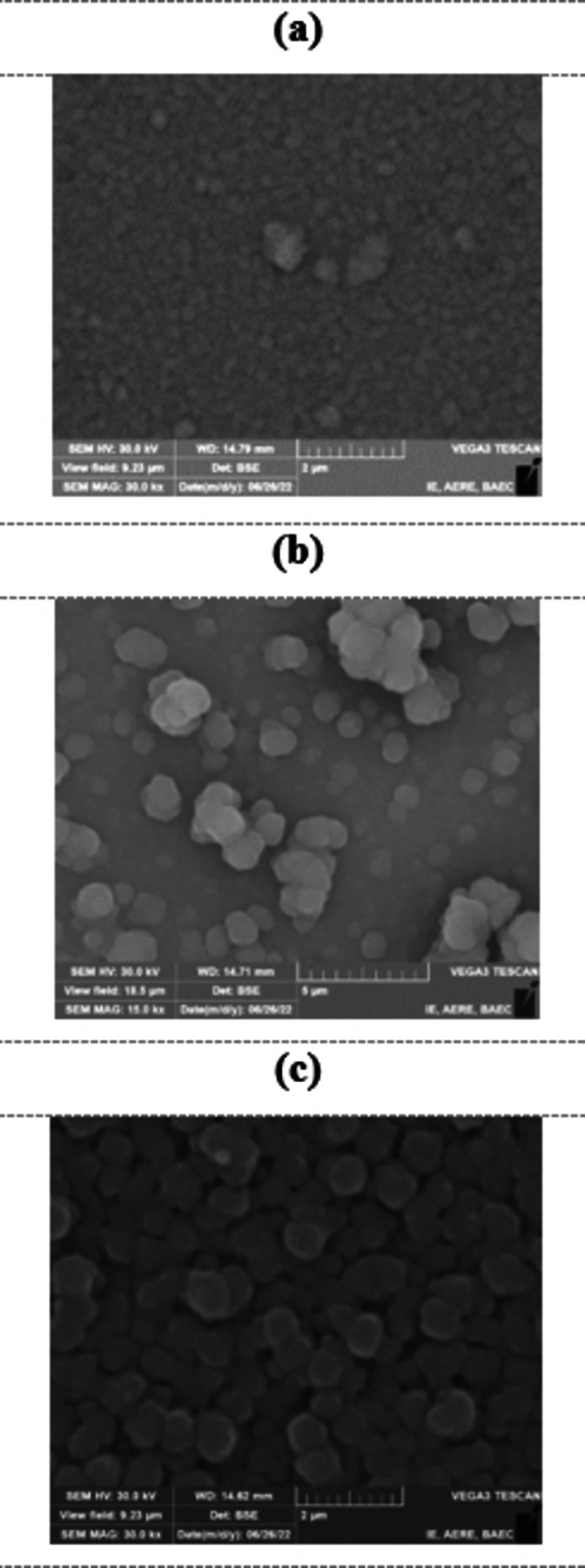
SEM images of CuI thin films deposited for (a) 5 min, (b) 7 min and (c) 10 min by HP‐PECVD method.

Figure 6 shows the surface morphology of CuI thin films prepared by HP‐PECVD technique. The figures show the SEM images of the CuI thin films with deposition time of 5, 7 and 10 min, respectively. It is observed from the images that the films are compact and continuous with the presence of some grains in the films. It is also apparent from the Figures that the grain size and film roughness in the films slightly increases with deposition time. Excess substances are deposited on the substrate as a result of the prolonged plasma interaction with the film. Besides, the substrate temperature increases as the deposition time increases. For example, for 3 min of deposition, the substrate temperature was raised to ~48.5 °C, whereas for 10 min, it was ~125 °C. As deposition temperature increases, atomic diffusion and surface mobility increases, causing grain growth to increase linearly, resulting in an increase in grain size. Plasma collisions and the subsequent thermal treatment are conducive to generating larger 3D spheres as a result of the presence of more atoms or molecules.[^40^, ^41^]

The compositional analysis of the CuI thin films deposited by HP‐PECVD method were done by energy dispersive spectrometry (EDS) studies. Table 1 shows the elements present in the films deposited for different time. The Table indicates that most of the particles found in the film were carbon. With increasing deposition time, the carbon percentage is gradually increased from 73.4 to 76.9 % for 5 to 10 min deposition. Nitrogen comes in the second position with 23.1 % for 5 min, 25.3 % for 7 min and 21.8 % for 10 min deposition. Percentage of copper is gradually decreased to 0.30 % from 0.70 %, whereas iodine is reduced to 1.10 % from 2.80 %, when the film deposition time is increases to 10 min from 5 min, respectively. As the deposition time increases, the mostly dominating carbon and nitrogen significantly increases. Therefore, the amount of copper and iodine decrease in the films. There is also possible to re‐evaporation of iodine due to the increase in film temperature due to prolonged exposure of plasma on the films.^[2]^ As a result, the stoichiometry of Cu and I may not be maintained in the HP‐PECVD‐deposited CuI thin films.

**Table 1:** The elemental composition of CuI thin films deposited by the HP‐PECVD method.

Sample	Carbon [wt %]	Nitrogen [wt %]	Copper [wt %]	Iodine [wt %]
5 min	73.4	23.1	0.7	2.8
7 min	71.9	25.3	0.5	2.4
10 min	76.9	21.8	0.3	1.1

**Figure 7:**
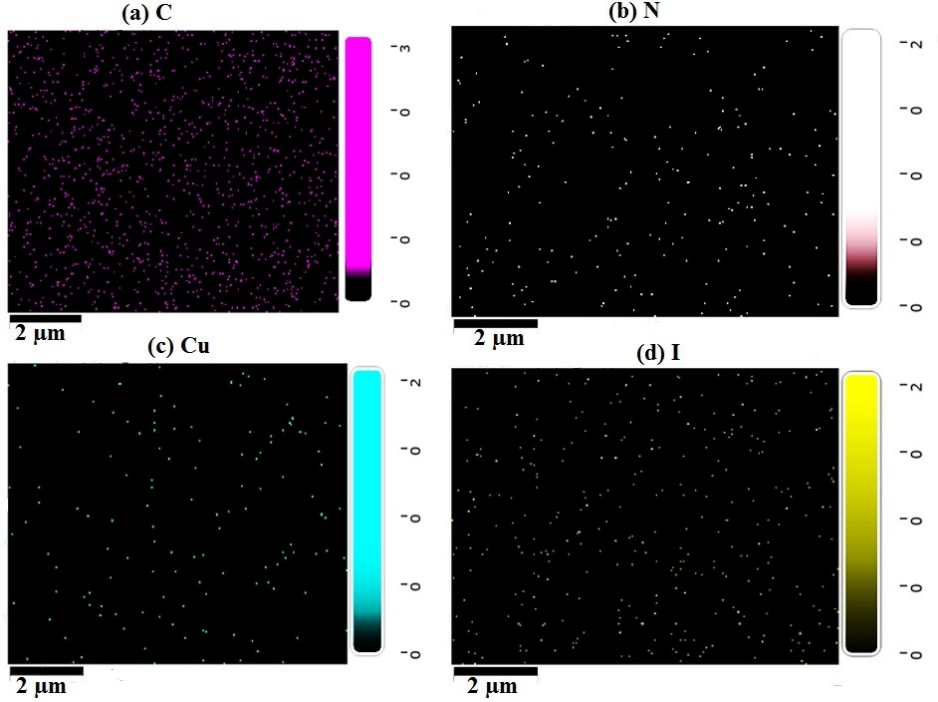
EDS mapping of CuI thin film showing presence of (a) C, (b) N, (c) Cu and (d) I, respectively for the deposition time of 5 min.

Figure 7 depicts the EDS‐based elemental mapping of the HP‐PECVD synthesized CuI thin films deposited for 5 min. The Figure reveals the presence of four elements C, N Cu and I, respectively. As also seen in Table 1, the carbon content is dense and dominating in the film. The Cu and I are discretely present in the film.

### FTIR studies of CuI thin films

**Figure 8:**
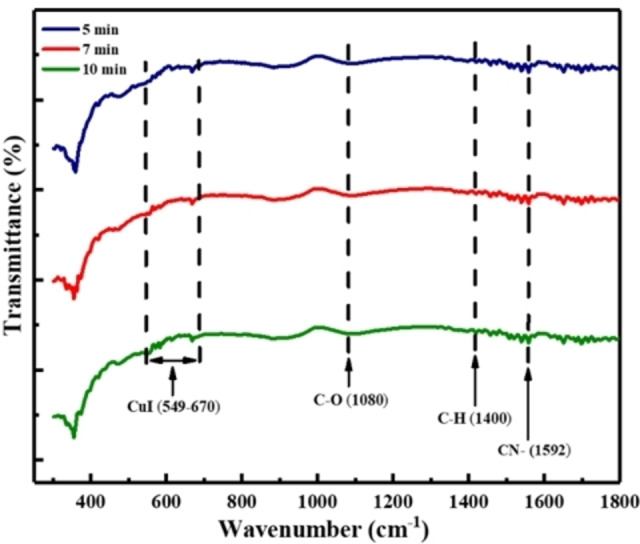
The FTIR spectra of CuI thin films deposited by HP‐PECVD method.

Figure 8 shows the FTIR spectra of CuI thin films deposited onto glass substrate by HP‐PECVD method in the range of 300–1800 cm^−1^. The absorption peak in the range of 549–670 cm^−1^ indicates the characteristic peak for Cu−I stretching vibrational bond.[^42^, ^43^] It is also observed from the Figure that the other peaks arrive at ~1080, 1400, 1592 cm^−1^ can be attributed to C−O, C−H and C=N stretching vibration bonds, respectively.[^42^, ^43^] No other peaks were detected in the range of 2000–4000 cm^−1^, therefore, not shown in the Figure. Therefore, HP‐PECVD method shows a potential technique for the deposition of low cost CuI thin films.

### Optical properties of HP‐PECVD CuI thin films

**Figure 9:**
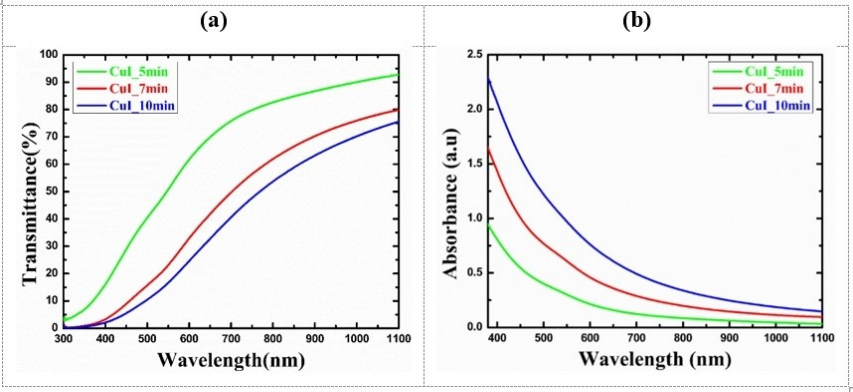
The (a) transmittance and (b) absorbance spectra of CuI thin films deposited by the HP‐PECVD method.

The optical properties of the synthesized CuI thin films were studied by measuring UV‐Vis spectra. Figure 9 shows the transmittance and absorbance spectra of CuI thin films deposited by HP‐PECVD method. It is visualized from the figures that the transmittance of the film is over 80–85 % in the range of 700–1100 nm for the film deposited for 5 min. The film deposited for 7 min shows a transmittance over 70–80 % in the range of 800–1100 nm which is consistent with earlier reports.[^2^, ^44^] As the deposition time increases, the transmittance of the films decreases which is expected as the thickness of the film increases with deposition time.

Likewise, the absorbance of the CuI films is lower for the film deposited for 5 min. After that, absorbance of the films increases with deposition time due to the increase in film thickness.

The optical band gap of the synthesized CuI thin films were measured from the Tauc plot of Equation 10,^[44]^

(10) $${\left(\alpha h\nu \right)}^{2}=A\left(h\nu -E_{g}\right)$$

where, α , A and $E_{g}$ represent the absorption coefficient, constant depending on the probability of transition and band gap, respectively.

**Figure 10:**
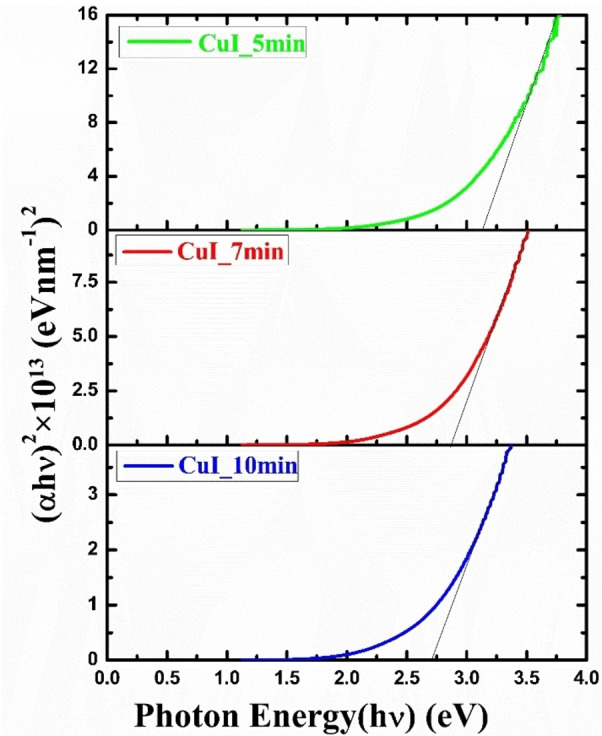
The Tauc plot of CuI thin films fabricated by HP‐PECVD method.

Figure 10 shows the optical band gap of the CuI thin films synthesized by HP‐PECVD method. It is observed from the Figure that the band gap of the CuI thin films decrease with the increase in deposition time, that is, thickness. It is observed from the EDS data that synthesized CuI thin film contains significant amount of C (over 70 %) and N (over 20 %) that increases with deposition time. These C and N act as dopants in the CuI thin films. Generally, these impurities create allowed energy states in the band gap and due to huge amount of impurities these will create a band near the valance band edge, therefore decrease the band gap of the CuI films.^[45]^ The band gap of the thin films may also vary owing to the variation of barrier height at grain boundaries with increasing thickness of the film. This may happened because of the rise in density of localized state near the band gap edges which in turn lower the band gap of the synthesized films.[^46^, ^47^] And also, the enhancement of the direct band gap of the CuI thin films owing to increase of thickness may be attributed to the augmentation of particle size, reduction of strain and increment in lattice constant.^[47]^ Therefore, despite the predominant presence of C and N in the films, it is CuI that governs the optical and electrical properties of the films. The band gap of the CuI films varies in the range of 2.71–3.14 eV for the deposition time of 5–10 min. These results are consistent with previous reports.[^43^, ^48^, ^49^] However, this change in band gap may be engineered for solving the problem of valance band offset in CuI/n−Si solar cell that would enhance the performance of the solar cells.[^50^, ^51^] Therefore, it can be concluded that higher transmittance and band gap engineering by changing the deposition time of the CuI thin films could be helpful in manufacturing low cost window and hole transport layers and hence low cost silicon and perovskite solar cells in future.

## Conclusions

This article demonstrates the production and characterization of CuI thin films for the application in low cost solar cells by HP‐PECVD plasma system. The power dissipated in the plasma was 13.60 W for C_2_H_3_N−CuI vapor. The XRD study revealed that the CuI films were polycrystalline in nature. The EDS mapping confirmed that the films prepared by this system mostly consisted of carbon followed by nitrogen, iodine and copper. The FTIR spectra of CuI thin films in the range of 549–670 cm^−1^ indicate the characteristic peak for CuI stretching vibration. Film deposition time played a vital role that significantly affects transmittance, absorbance and optical band gap energy. The transmittance of 80–85 % was found in the range of 700–1100 nm for the films deposited for 5 min. The band gap of the films varied in the range of 2.71–3.14 eV depending on the deposition time which would be helpful for the fabrication of low cost p‐type window and hole transport layers and hence cost efficient silicon and perovskite solar cells.

## Conflict of interest

The authors declare no competing financial interest.

1

## Data Availability

The data associated with the experiment are available free of charge from authors upon reasonable request.
